# Neural basis of attachment-caregiving systems interaction: insights from neuroimaging studies

**DOI:** 10.3389/fpsyg.2015.01241

**Published:** 2015-08-24

**Authors:** Delia Lenzi, Cristina Trentini, Renata Tambelli, Patrizia Pantano

**Affiliations:** ^1^Dipartimento di Neurologia e Psichiatria, Univeristà SapienzaRome, Italy; ^2^IRCCS San Raffaele La PisanaRome, Italy; ^3^Centro di Terapia Metacognitiva InterpersonaleRome, Italy; ^4^Dipartimento di Psicologia Dinamica e Clinica, Univeristà SapienzaRome, Italy; ^5^IRCCS NeuromedRome, Italy

**Keywords:** attachment, caregiving system, limbic system, fMRI, orbitofrontal cortex, amygdala, trauma, AAI

## Abstract

The attachment and the caregiving system are complementary systems which are active simultaneously in infant and mother interactions. This ensures the infant survival and optimal social, emotional, and cognitive development. In this brief review we first define the characteristics of these two behavioral systems and the theory that links them, according to what Bowlby called the “attachment-caregiving social bond” (Bowlby, 1969). We then follow with those neuroimaging studies that have focused on this particular issue, i.e., those which have studied the activation of the careging system in women (using infant stimuli) and have explored how the individual attachment model (through the Adult Attachment Interview) modulates its activity. Studies report altered activation in limbic and prefrontal areas and in basal ganglia and hypothalamus/pituitary regions. These altered activations are thought to be the neural substrate of the attachment-caregiving systems interaction.

## The Attachment System and the Caregiving System

Attachment theory (Bowlby, 1969) postulates that humans are born with a psycho-biological system that motivates them to seek proximity to significant others (in particular, the mother) in time of need, with the aim of acquiring a feeling of security. This system includes a variety of non-verbal expressions of neediness and desire for proximity such as crying and looking, as well as active approaching behaviors which aim to reestablish and maintain proximity, such as moving toward the caregiver and clinging (Ainsworth et al., 1978).

Bowlby (1969) also delineated the provisions that the mother should guarantee: proximity maintenance, when the child is in time of need; a physical and emotional safe haven, in which infant’s distress may be alleviated; she should acts as a secure base, from which the child may face the outside world and to which he/she may return with the sense of being comforted if distressed and reassured if frightened.

Bowlby (1969, 1988) proposed that caregiving is the result of an organized behavioral system, which is reciprocal to – and evolved in parallel with – the attachment system (George and Solomon, 1996, 1999). The caregiving system aim is to promote proximity and comfort when the mother detects internal or external cues associated with situations that she perceives as stressing for the child.

In women, this system remains immature until late adolescence. During puberty and in late adolescence hormonal and neurobiological changes interact with environmental stimuli and prior attachment experiences (George and Solomon, 1996, 1999; Ammaniti et al., 2000; Grossmann et al., 2005) to push the caregiving system toward maturity.

The maternal caregiving system undergoes its greatest development during the transition to parenthood (pregnancy, birth, and the post-partum period; Ammaniti et al., 2014) with striking structural and functional changes, as a result of the large amounts of hormones secreted (Panksepp, 1998; Mayes et al., 2005). In particular, of greatest importance is the production of oxytocin which seems to motivate and maintain caregiving behaviors, strengthening maternal sensitivity to infant affective cues (Frewen and Lanius, 2006; Kinsley and Lambert, 2006; Rilling, 2013; Mah et al., 2015).

## Affect Regulation and Attachment

A mother’s capacity to regulate her child’s emotions is crucial to his/her ultimate feeling of security (Ainsworth et al., 1978; Lyons-Ruth and Spielman, 2004). These processes are sustained by maternal sensitivity, i.e., the ability to understand the infant’s feelings in order to respond to them in an appropriate way (Ainsworth, 1967, 1973; Ainsworth et al., 1978).

Fonagy et al. (1991a) have suggested that these sensitive responses are guided by maternal reflective functioning (RF) that is the capacity to ascribe the baby mental states (intentions, motivations, and feelings).

Early affective experiences are progressively internalized as internal working models (IWMs), which can be regarded as generalized representations of “lived experiences” (Bretherton et al., 1986; Bretherton, 1987), prototypical representations of the other and of the self, since they contain information about whether the attachment figure is perceived as a person who responds to calls for support or protection, and if the self is worthy of receiving help (Bowlby, 1969, 1973).

Repeated interactions with mothers who are emotionally available and sensitive facilitate the optimal functioning of the child attachment system, and promote the development of attachment security (Bowlby, 1973, 1988). Moreover, positive expectations about others’ availability and positive representations of the self as competent and valued are formed, and affect-regulation strategies are organized around these positive beliefs.

Viceversa, when the mother proves not to be physically or emotionally available security is not attained and negative representations of the self and the other are formed (e.g., doubts about self-worth and worries about others’ intentions). As a result, two strategies of affect regulation other than proximity seeking are likely to be adopted: *deactivation or hyperactivation* of the attachment system.

Deactivating strategies are used as “flight” reactions from a mother who is seen as emotionally unavailable (Main and Solomon, 1990). The child learns to hide or suppress the expressions of emotions that the mother does not tolerate (anxiety, fear, anger, or needs of consolation) and deals with threats and dangers autonomously, to avoid the frustration caused by maternal unavailability.

Conversely, hyperactivating strategies represent “fight” responses to unfulfilled attachment needs, acted when maternal responsiveness appears inconsistent, hesitant, or unpredictable (Mikulincer and Shaver, 2010): the child tends to amplify proximity seeking strategies to demand or force the mother to pay more attention to him/her (Main and Solomon, 1990; Mikulincer and Shaver, 2010).

## Examining Individual Differences in the Attachment System: Attachment Models

Attachment models reflect ones’ most accessible IWM and the typical functioning of ones’ attachment system.

For early childhood, the Strange Situation Procedure (SSP; Ainsworth et al., 1978) is the most widely used to assess patterns of individual difference in attachment. By exposing infants to increasing challenges to the attachment system (i.e., the presence of a strange person and two short separations from the mother), the SSP originally classified infants in three categories: secure (tipe B, indicating successful proximity-seeking attempts and security attainment); insecure avoidant (type A, characterized by deactivating strategies); or insecure anxious-ambivalent (type C, characterized by hyperactivating strategies). Main and Solomon (1990) later added a fourth category, “disorganized/disoriented,” defined by odd, awkward behavior and unusual fluctuations between anxiety and avoidance.

Internal working models are thought remain fairly stable throughout one’s lifespan, guiding the individual’s functioning and the construction of significant relationships, particularly parental one (Bowlby, 1988; Shaver and Mikulincer, 2002; Cassidy and Shaver, 2010)

Adopting a developmental and clinical approach, Main and Goldwyn (1984) developed the Adult Attachment Interview (AAI), which evaluates adults’ mental representations referred to attachment relationships. AAI can be also coded in accordance with the Dynamic Maturational Model (DMM) of Attachment and Adaptation (Crittenden and Landini, 2011).

In the AAI, adults are asked to retrieve attachment-related autobiographical memories from early childhood and to evaluate these memories and their effects from their current perspective. In this way what is coded is the structural dimension of the transcript (that is, its “coherence” or “incoherence”) and not its content. The classical AAI coding system classifies adults into three major categories, paralleling Ainsworth’s infant typology: secure/autonomous with respect to attachment (F); Dismissing of attachment (Ds); Preoccupied with or by early attachments or attachment-related experiences (E). In the presence of unresolved responses regarding experiences of loss or trauma, transcripts can receive the additional classification of Unresolved/disorganized (U/d). Finally, when texts cannot be fitted to any organized AAI placement, the classification Cannot Classify (CC) is applied. Conversely, with the DMM, patterns of attachment are considered to be ‘self-protective strategies’ that varied dimensionally (rather than categorically) in terms of the use of cognitive-contingent or affect-arousing information. Moreover, each individual is thought to have multiple “dispositional representations” that regulate behavior under different conditions. One novel construct within the DMM coding of the AAI is “reorganization,” a process whereby speakers are actively changing their understanding of past and present experiences and moving toward attachment security.

The use of the AAI provided significant evidence for the intergenerational transmission of attachment, allowing the investigation of the dynamics through which IWMs (and its expression through the caregiving system) influence the child’s attachment development (Main et al., 1985; Fonagy et al., 1991b).

Secure individuals have had infantile experiences with parents who guaranteed protection and emotional availability toward their attachment needs. They have worked out childhood relationships and recognize its relevant value for their-own personal history and their current mental state. When these individuals become parents, this personal orientation enables them to respond affectionately to their child’s demands for safety. Thus, the child will internalize a feeling of security and relational trust.

Dismissing subjects, on the other hand, have had infantile experiences of refusal toward their emotional needs. They seem incapable of valuing their attachment relationships, they find it difficult to remember early relational experiences, and they do not show affective responses to their memory of early and painful situations. In such cases, defensive mechanisms of splitting and denial are used, in order to compensate for the affect dysregulation resulting from painful autobiographical memories, and maintain an idealized vision of the self and of others. The same defensive style will be noticeable in their children, who will tend to escape from self-involving affective interactions.

Preoccupied individuals seem incapable of de-identifying themselves from their own childhood relationships, since they are entangled in worried and angry feelings about parents. They appear hypersensitive to attachment experiences, and can easily retrieve negative memories but have trouble discussing them coherently without anger or anxiety. Children with preoccupied caregivers frequently show marked ambivalence toward them, since they seek a relationship and, at the same time, express anxiety, fear, and anger.

Individuals classified as unresolved are disoriented in their discussion about their childhood history of loss or trauma, as indicated by lapses in monitoring reasoning or discourse (Main and Hesse, 1990; Hesse and Main, 2000); moreover, their emotion regulatory strategies reflect a lack of resolution of these life events (Main and Hesse, 1990). Children of individuals classified as “unresolved” frequently show disorganized attachment, appear frightened and alarmed showing immobilized behavior and dazed appearance (Van IJzendoorn, 1995) caused by the caregivers’ failure in monitoring children’s behavior during interactions, and in regulating their signals of distress.

## Insights from Neuroimaging Studies

In recent decades neuroscientists have been trying to investigate the neural bases of attachment and caregiving systems in humans mainly by using neuroimaging techniques functional magnetic resonance, (fMRI), enabling them to study the brain “in action” during different tasks.

Several fMRI studies have addressed these two systems. Those which have explored the attachment system have found activity in various areas, among which amygdala, anterior cingulum, stria terminalis, preoptic area, and basal ganglia (Bartels and Zeki, 2004; Gillath et al., 2005; Lemche et al., 2006; Coria-Avila et al., 2014). Partially overlapping areas have also been found to be related to the activation of the caregiving system, i.e., limbic and para-limbic areas, basal ganglia, medial prefrontal areas (orbitofrontal cortex and anterior cingulum) and midbrain nuclei (Leibenluft et al., 2004; Nitschke et al., 2004; Swain, 2008; Laurent and Ablow, 2012). So far, only a small body of research with fMRI has examined how the maternal attachment model affects the activity in brain areas during the activation of the caregiving system. Therefore, we will present scientific literature on the interaction between these two systems, by examining those experiments which have exposed women to infant stimuli activating the caregiving system and have studied the effect of attachment model on their brain activation (see **Table 1**).

**Table 1:** Summary of fMRI studies on attachment-caregiving systems interaction.

Study	Task	Stimuli	Attachment Measure	Subjects	Analysis	Results
Kim et al. (2014)	Viewing	Own and other child pictures	AAI (DMM)	42 Mothers: 25 No trauma, 17 U, Unresoved trauma.	ROI in bilateral amygdale	No trauma or loss >*U* • Bil amygdala
Riem et al. (2012)	Listening	Crying child sound and control sound	AAI	21 Nulliparous women: 7 *F*, 4 *D*s, 4 *E*, 6 *U*.	ROI in bilateral amygdale	*D*s e *E* > *F* (crying > control sound): • R amygdala Negative correlation: R Amygdala and Coherence of Mind
Strathearn et al. (2009)	Viewing	Own and other child pictures	AAI (DMM)	30 Mothers: 15 *F*, 15 *D*s.	Whole brain and ROI in midbrain, striatum, prefrontal cortex (PFC), hypothalamus/pituitary regions	*F* > *D*s: • Bil frontopolar PFC • Bil orbitofrontal and medial PFC • L ventral striatum • L hypothalamus/pituitary region *D*_s_ > *F*: • Dorsolateral PFC • Medial PFC • R uncus/enthorinal cortex. • Bil insula • R anterior cingulate cortex Direct correlation: Hypothalamus/pituitary/ventral striatum and peripheral oxytocin responce
Lenzi et al. (2013)	Empathizing	Child pictures	AAI	23 Nulliparous woman: 11 *F*, 12 *D*s	Whole brain	*D*_s_ > *F*: • Bil somatomotor and premotor cortex, inferior frontal gyrus • R superior frontal gyrus • R superior temporal sulcus, temporal pole, hippocampus • L middle temporal gyrus, • L anterior cingulum • L posterior parietal cortex • Bil thalami • Bil precuneus *F > D_s_ (deactivation in D_s_):* • medial orbitofrontal cortex • perigenual anterior cingulated cortex.
Lenzi et al. (2009)	Empathizing	Own and other child pictures	RF (AAI)	16 Mothers: 13 *F*, 3 *D*s	ROI	Direct correlation: Reflective Function with R anterior insula


*F,secure/autonomous with respect to attachment; Ds, dismissing attachment; E preoccupied with or by early attachments or attachment-related experiences; U, unresolved with respect to attachment trauma; DMM, coding method based on the Dynamic Maturational Model of Attachment and Adaptation; RF, reflective function; ROI, region of interest, R, right; L, left*.

To start we will briefly provide a description of the results obtained in these studies and then we will discuss common and discordant findings in the background of current theories on attachment and caregiving system.

The first study focusing on this issue was that of Strathearn et al. (2009). They examined 30 mothers and the difference in their reaction to exposure to pictures of crying, smiling and neutral faces of both their own and other children. By doing so they were able to test whether differences in attachment were related to brain reward areas activation and peripheral oxytocin response to infant cues. In this study authors focused on specific areas, i.e., the midbrain, striatum, prefrontal cortex (PFC) and the hypothalamus and found that mothers with secure attachment greatly activated for the own infant the frontopolar PFC bilaterally, the ventral striatum, and the oxytocin-associated hypothalamus/pituitary regions. Positively, activity in these last two regions was significantly higher in secure mothers, and correlated with peripheral oxytocin response to infant contact. Conversely, dismissing mothers greatly activated other parts of PFC, i.e., the dorsolateral and medial PFC, including the anterior cingulate cortex, as well as the uncus/enthorinal cortex. These results are in line with the finding that maternal plasma oxytocin concentrations are positively correlated with affectionate behavior toward the child, who (in turn) responds to this affection with positive parent-directed behaviors (Rilling and Young, 2014).

A second study by Riem et al. (2012) focused a priori on the activity of the amygdala in a group of 21 nulliparous women listening to infant crying. They found that those who had been classified as insecure (specifically *D*s and *E* coded subjects) greatly activated the amygdala when compared to secure women. Coherently, the amygdala activity was negatively correlated with coherence of mind scores.

The important role of the amygdala was confirmed in a further study on mothers with unresolved trauma, who showed reduced bilateral amygdala response when viewing their own infants’ sadness, when compared to happiness, with respect to mothers with no trauma (Kim et al., 2014).

**FIGURE 1:**
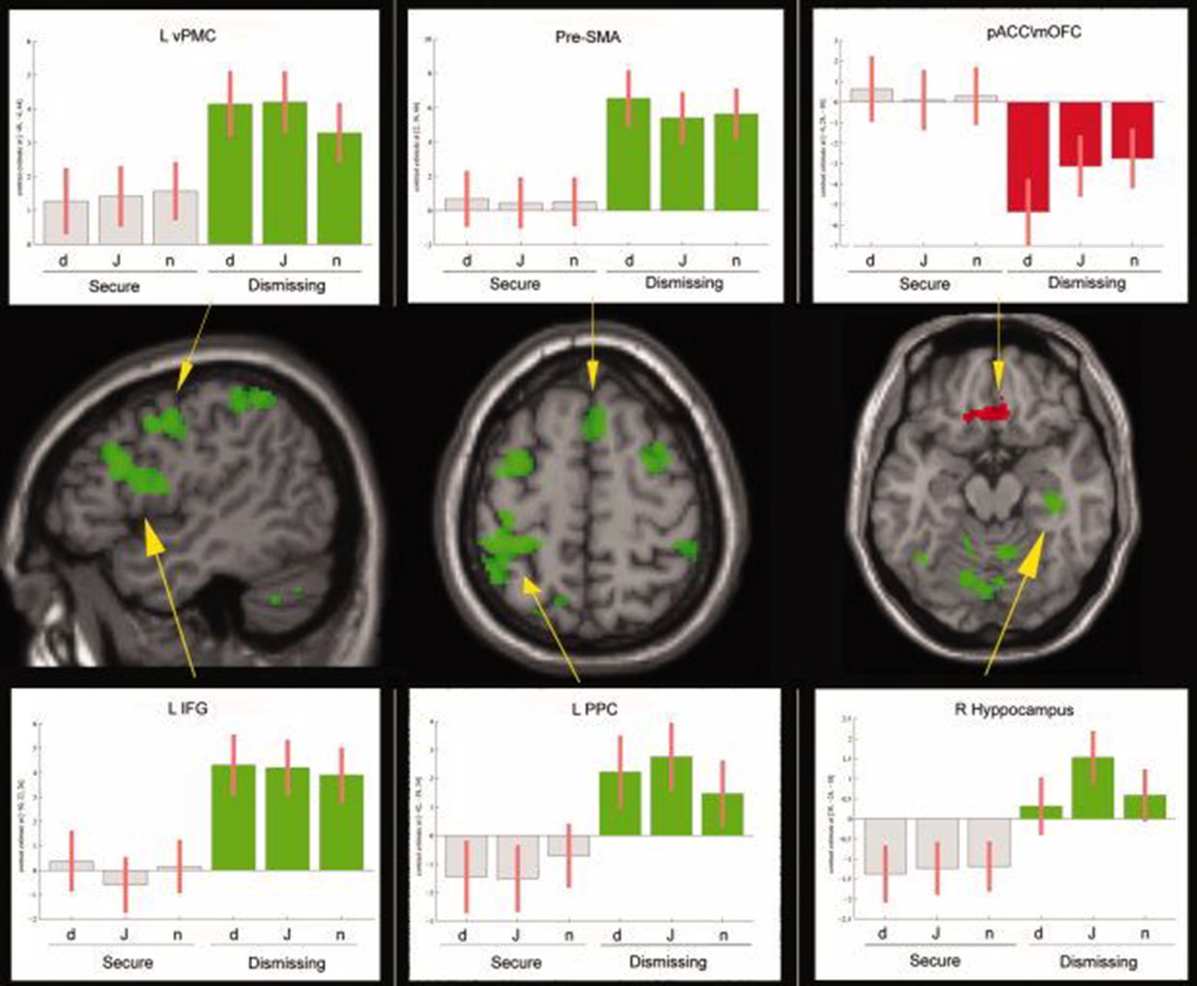
**From Lenzi et al. (2013)**. Empathizing task, *F* > *D*s and *D*s > *F* contrasts reported on the SPM *T*_1_–WI standard template sections. Areas in green are those more active in *D*s than in *F*. In red are shown those areas less active in *D*s than in *F* (contrast *F* > *D*s). For some areas we also show the corresponding signal plot. MNI coordinates are shown in brackets. All statistical maps are projected at a threshold of *P* < 0.001 uncorrected, corrected at the cluster level *P* < 0.05. a.u., arbitrary units, 90% confidence interval (C.I.); d, distress; j, joy; n, neutral; R, right; L, left; pre–SMA, presupplementary motor area; vPMC, ventral premotor cortex; IFG, inferior frontal gyrus; PPC, posterior parietal cortex; pACC/mOFC, pregenual anterior cingulate cortex, and medial orbitofrontal cortex. *Reproduced with permission*.

Lastly, two other experiments, both by our group, have explored this field of research. In the first one (Lenzi et al., 2009) we examined 16 mothers with fMRI while observing/empathizing faces of their own child and those of someone else’s child and found that the right anterior insula activity was directly correlated with maternal reflective function. In the second study (Lenzi et al., 2013) we studied a group of nulliparous women either with secure or dismissing model. Analysis revealed that dismissing women activated to a significantly greater extent in respect to secure ones several areas, i.e., frontal areas (bilateral somatomotor and premotor cortex, inferior frontal gyrus, left anterior cingulate cortex, and right superior frontal gyrus), temporal (right middle temporal gyrus, superior temporal sulcus and the right hippocampus and temporal pole), parietal (left posterior parietal cortex and bilateral precuneus). Moreover, the medial orbitofrontal cortex and the perigenual part of the cingulate cortex were more deactivated in dismissing women (**Figure 1**).

## Discussion

It is worth acknowledging that in all of the aforementioned studies the attachment model has been coded with the AAI, according to the main literature in this field that consider it to be the gold standard method for exploring adult representation of attachment (i.e., IWMs). In particular Strathearn et al. (2009) and Kim et al. (2014) used the DMM as AAI coding system (Crittenden and Landini, 2011) whereas the other groups used the classic method described by Main and Goldwyn (1984). It is also worth keeping in mind that in two studies researchers studied nulliparous woman (Riem et al., 2012; Lenzi et al., 2013) whereas in the other they focused on mothers. This information is important because it could explain, at least in part, some of the different results found in these experiments.

An important data that emerge is that the limbic/paralimbic network seems to play an important role in the interaction between attachment and caregiving systems (**Table 1**). In particular data consistently showed within this network altered activation of the amygdala, the hippocampus, the uncus/entorinal cortex, the temporal pole and anterior cingulate cortex. Increased activity in all these areas has been found in most studies in dismissing and preoccupied, as compared to secure subjects (Lenzi et al., 2009, 2013; Strathearn et al., 2009; Riem et al., 2012). One study though reported reduced activity in the amygdala in mothers with unresolved trauma with respect to those without unresolved trauma (Kim et al., 2014).

Increased activation in limbic and paralimbic areas in insecure subjects is thought to represent the neural correlate of affective dysregulation possibly due to the reactivation of infantile memories of parental rejection toward their own attachment needs. This leads to negative experiencing of infant cues and of negative internal attribution to the nature of the infants signs of distress. This emotional dysregulation is also supported by our findings of increased activity in empathy-related areas, i.e., mirror areas (premotor cortices, inferior frontal gyrus), in dismissing women when observing/empathizing with infant faces (Lenzi et al., 2013).

Conversely, and apparently in contrast with other results, is the reduced response of the amygdala in subjects with unresolved trauma. Such reduced activation of the amygdala found by Kim et al. (2014) has been interpreted as emotional suppression to protect the mothers with unresolved attachment-related trauma from re-experiencing traumatic memories, akin to the so called “defensive numbing” that develops upon continued traumatization (Bowlby, 1988; Liotti, 2006).

Greater activity in the prefrontal cortices, basal ganglia, and hypothalamus/pituitary regions has been reported in secure women with respect to organized dismissing and preoccupied women (Strathearn et al., 2009; Riem et al., 2012; Lenzi et al., 2013) The greater activity in prefrontal areas, in particular orbitofrontal cortex and lateral PFC, likely represents the expression of increased activation of the attachment system. The basal ganglia increased activation suggests the involvement of the reward system, in line with the hypothesis that for securely attached women infant cues are salient signals able to reinforce and motivate the activation of the caregiving system. Last but not least is the finding of greater activity in secure women in oxytocin-associated areas, i.e., the hypothalamus/pituitary region, known to be strictly involved in promoting and maintaining maternal behavior (Rilling and Young, 2014). Activation of reward and oxytocin-associated brain areas is probably the substrate of the activation of a sensitive and efficient caregiving system, able to promote a secure attachment model in the offspring.

There are other contrasting results worth mentioning, i.e., the insula is more active in dismissing mothers in respect to secure mothers (Strathearn et al., 2009) but is also directly correlated to reflective function (Lenzi et al., 2009). These two studies were different in terms of aims and methods (in the second there was only one group of mothers and the aim was studying own versus other child neural response) but further studies focusing on the anterior insula should be conducted in order to explain the role of this area in the attachment and caregiving systems interaction.

## Conclusion and Implications for Future Research

Research on neural bases of attachment-caregiving system interaction is still in its infancy and additional data is needed to confirm these findings in larger cohorts of women, possibly simultaneously including all of the different attachment models. Current fMRI literature is in line with data coming from clinical research on attachment suggesting emotional dysregulation and disturbed maternal caregiving in insecure organized women when compared to secure subjects and emotional numbing in those with unresolved trauma.

## Conflict of Interest Statement

The authors declare that the research was conducted in the absence of any commercial or financial relationships that could be construed as a potential conflict of interest.
